# Trends and Prospects of Genetic and Molecular Research in Plants

**DOI:** 10.3390/plants13111545

**Published:** 2024-06-03

**Authors:** Yu Jin Jung, Me-Sun Kim, Yong-Gu Cho

**Affiliations:** 1 Division of Horticultural Biotechnology, School of Biotechnology, Hankyong National University, Anseong 17579, Republic of Korea; 2 Institute of Genetic Engineering, Hankyong National University, Anseong 17579, Republic of Korea; 3 College of Agriculture and Life & Environment Sciences, Chungbuk National University, Cheongju 28644, Republic of Korea; kimms0121@cbnu.ac.kr (M.-S.K.); ygcho@cbnu.ac.kr (Y.-G.C.)

With the exponential advancements in biotechnology research, various studies are being conducted to overcome productivity limitations in crop breeding. Climate change, a consequence of ozone layer destruction, acts as the greatest stress on plants, affecting the growth and development of crops, ultimately leading to a decrease in production and threatening food security [1]. On the other hand, the world’s population is increasing exponentially, approaching 9 billion, so research to increase crop productivity is essential. Therefore, it is not only very difficult to meet food demand using traditional breeding methods that have been used in the past, but it is also time-consuming and impossible to manage [2,3]. Crop breeders will be able to overcome the difficulties they face by utilizing various breeding methods based on plant physiology, genetics and molecular research accumulated so far. Recently, in crop breeding, the development of omics and gene editing technology has made it possible to precisely manipulate target genes, enabling the breeding of crops with the characteristics desired by researchers [4]. As a representative example, plant protein production has become possible based on transcriptome and metabolite research, genome editing, and functional material research. Therefore, in the future, multifaceted efforts should be made to develop breeding technology and improve crops to provide safe and secure food and pharmaceutical raw materials to humanity.

This Special Issue of *Plants* is a continuation of numerous optimistic applications of genomics to enhance food crop production, based on detailed knowledge of genetic structure and its intergenomic interactions not observed in food production environments. Genomics can provide important data for food production in three ways. First, genomics can be utilized as an important element in breeding programs as a powerful tool for identifying and characterizing plants of commercial interest. Second, genomics can be utilized as a tool to monitor the impact of plants or microorganisms on the environment, and to adjust the growth conditions they require. Third, plants or microorganisms can contribute to the development of new varieties with improved agricultural, health, nutritional, or processing characteristics by utilizing biotechnology. Therefore, this Special Issue encompasses manuscripts reporting on important data that are utilized as various tools for food production.

These manuscripts comprise 1 review and 16 research papers, including epigenetics and plant-protein-related gene function studies, genome analysis studies, transcriptome and metabolome studies, gene editing, and crop stress-related studies. Articles on this topic focus on various biotechnological studies aimed at improving crop productivity due to climate change.

This Special Issue includes two articles that performed transcriptome analysis. In the first, Cho et al. [5] revealed correlations between ncRNAs and target coding RNAs through transcriptome analysis of suspension cells derived from damask rose petals, revealing important biological molecular mechanisms that occur during cell culture. In the second article, through an integrated analysis of the metabolome, transcriptome, and physiology, Lai et al. [6] found that extensive metabolic and genetic reprogramming occurred in N-deficient roots, which not only improved the ability to detoxify reactive oxygen species and aldehydes, but also delayed root aging due to oxidative damage. 

Additionally, four articles are dedicated to epigenetic changes and plant proteins. Pírek et al. [7] used a mass spectrometry-based approach to investigate epigenetic changes in histone proteins during callus formation in the roots and shoots of *Arabidopsis thaliana* seedlings. The results showed that an early callus derived from seedling shoots and roots showed specific epigenetic patterns, whereas a long-term cultured callus was homogenized. This phenomenon emphasized the importance of HDAC inhibitors in the culture process of calluses. Antonio et al. [8] cloned a putative gene corresponding to RPAP3, which plays the same role as the chaperone R2TP, in the monocot *Sorghum bicolor*, characterizing the SbRPAP3 protein with 396 residues. As a result, SbRPAP3 not only maintained its functions, including binding to RUVBL, Hsp90, and Hsp70, but also showed that RPAP3 performs an important function in the R2TP complex. In the third article, Pei et al. [9] identified 30 PITP (phosphatidylinositol transfer protein)-related genes in the rice genome, and investigated their characteristics, including physicochemical properties, gene structure, conserved regions, and subcellular location. The promoter region of the *OsPITPs* gene contained at least one type of hormone response element, such as methyl jasmonate (Me JA) and salicylic acid (SA). Additionally, the expression levels of OsPITP-related genes were found to be greatly affected by *Magnaporthe oryzae* infection. In their review article, Chuong et al. [10] reported that N6-adenosine methylation (m^6^A) is an evolutionarily conserved, dynamic, and reversible process across species that occurs as a complex with a variety of proteins, including methylases, demethylases, and m^6^A binding proteins. In plants, the regulatory function of the m^6^A methylation component has been shown to be involved in various elements of RNA processing, such as RNA stability, alternative polyadenylation, and miRNA regulation. The authors also summarized the molecular mechanisms behind m^6^A modifications and recent insights into the diverse roles of m^6^A in RNA regulation. 

Recently, whole-genome resequencing studies were performed in a variety of plants, including rice and soybean. In addition, whole-genome rearrangement-based InDel markers are attracting more attention due to their advantages such as wide distribution in the genome, stable mutations, and easy detection. This Special Issue presents five articles related to genomic analysis and markers. Shim et al. [11] investigated the characteristics of 6 types of plants from 220 types of milk thistle (*Silybum marianum* L. Gaertn.) resources collected at home and abroad, and 177 types of InDel markers were selected from 6 types of resources representing genetic diversity. This revealed the possibility of using milk thistle genetic research and breeding programs. Li et al. [12] evaluated the frequency of large-effect recessive mutations in tomatoes. To investigate the potential causes of recessive inherited resistance, 10 susceptibility-related S genes (*PMR 4*, *PMR5*, *PMR6*, *MLO*, *BIK1*, *DMR1*, *DMR6*, *DND1*, *CPR5*, and *SR1*) were selected. The results showed that three genotypes carrying homozygous SNPs with a significant effect on the S gene were infected with *Oidium neolycopersici*, and two had significantly reduced susceptibility to the fungus. Sgaramella et al. [13] used SNP markers to genotype a segregating population of recombinant inbred lines (RILs) obtained by crossing the Ethiopian Purple line of durum wheat with modern durum cultivars. Additionally, the total anthocyanin content (TAC), grain color, and L*, a*, and b* color indexes of whole wheat flour were evaluated in four environments. Therefore, the authors concluded that the obtained TAC and grain pigment-related identification markers will support marker-assisted selection (MAS) in durum wheat breeding programs aimed at improving the quality of durum wheat grains and derived foods. Choi et al. [14] amplified and sequenced 83 soybean mosaic virus (SMV) CP (envelope protein) sequences collected from soybean plants exhibiting severe disease symptoms from seven regions in Korea. The results revealed the geographic region or plant host as the most important factor for the genetic diversity of 305 SMV CP sequences. For genome size and ploidy level diversity among the Cannabis (*Cannabis sativa* L.) species, Balant et al. [15] examined genome size diversity for 483 individuals belonging to 84 species, including numerous wild/feral, landrace, and cultivated species, covering a wide range of distributions. Additionally, the authors performed sex determination using the MADC2 marker, and investigated the potential of flow cytometry as a method for early sex determination. All individuals were diploid, with genome sizes ranging from 1.810 to 2.152 pg/2C (1.189-fold variation), except for triploid at 2.884 pg/2C. The results suggest that the geographic expansion and domestication of cannabis had little effect on the overall genome size. 

To overcome crop productivity and efficient breeding amid the rapid climate change crisis, gene function research and gene editing, including plant stress, are considered research strategies that must be conducted and developed. This Special Issue contains six articles on functional studies of plant stress-related genes and gene editing. Wang et al. [16] showed that the expression of the cryptochrome function inhibitor *BIC2* gene, a regulator of ABA response in *Arabidopsis*, was significantly increased upon ABA treatment. Moreover, the expression of genes related to ABA signaling was found to be significantly affected in *BIC2*-overexpressing transformants and mutant *bic2*. Acevedo et al. [17] showed that the survival rate was increased after NaCl treatment in tobacco transgenic lines of the *DcPSY2* gene, and the expression of abiotic stress-related genes and ABA-related genes were upregulated in roots. Therefore, a direct correlation was noted between endogenous carotene production and an increased expression of abiotically relevant genes. Hussain et al. [18] isolated and characterized two *ABA-induced serine-rich repressor 1* (*ASR1*) and *ASR2* genes related to hitherto unknown ABA response genes. These genes not only contain a lot of serine in the middle region (MR) of the auxin response factor (ARF) repressor, but also contain the *ASR1* gene-specific L × L × L EAR motif. In *Arabidopsis thaliana*-overexpression transgenic plants and gene-edited mutants, *ASR1* and *ASR2* are negatively regulated redundantly in response to ABA. Salvador et al. [19] investigated how these19 *sHSP* genes were regulated during ripening and after nitric oxide (NO) gas treatment in pepper fruit transcriptome (RNA-Seq) analysis. The time course expression analysis of these genes during fruit ripening showed that six genes were upregulated and seven genes were downregulated, while six genes were not significantly affected. Additionally, NO treatment caused the upregulation of seven *sHSP* genes and downregulation of three *sHSP* genes, while nine genes remained unchanged. The authors emphasized that *sHSPs* are important for stress tolerance and that the observed changes in *sHSP* expression relate pepper fruit ripening to physiological nitro-oxidative stress. Pfotenhaurer et al. [20] used CRISPR/Cas9 to generate potato lines in which the *FtsZ1* gene, a tubulin-like GTPase that regulates plastid division, was edited. As a result, the starch granule size of the tubers increased 1.98-fold compared to the control, and the most promising edited line increased the final viscosity of the starch paste 2.07-fold compared to the control. Kim et al. [21] used the CRISPR/Cas9 system to generate an orange (OC) rice callus line through the targeted mutagenesis of the *OsOr* gene. In cell lines with edited *Or* genes, lycopene, β-carotene, and two β-carotene isomers were significantly increased compared to the control, showing improved total carotenoid content. 

Finally, this Special Issue aimed to cover a variety of biotechnological research to overcome the limitations of crop productivity by collating a diverse collection of interesting studies and review articles. We believe that the articles published in this Special Issue will play an important role in providing resources and knowledge to many readers, thereby stably increasing crop yields for future food security and developing valuable breeding resources.
